# Supplementary material for: Investigating Adherence to COVID-19 Vaccination and Serum Antibody Concentration among Hospital Workers—The Experience of an Italian Private Hospital
Source: Vaccines (Basel). 2021 Nov 16;9(11):1332. doi: 10.3390/vaccines9111332 (PMC8625988; doi:10.3390/vaccines9111332)
Supplement: Supplementary file 1 [file vaccines-09-01332-s001.zip › vaccines-1441831-supplementary.pdf]

SURVEY
Risposta anticorpale e aderenza campagna vaccinale

OBIETTIVO DELLA SURVEY

Indagine volta a determinare la risposta anticorpale a seguito della somministrazione del vaccino a operatori sanitari e non dell'Istituto Fiorentino di Cura e Assistenza (IFCA) S.p.A. Inoltre, si vuol valutare il grado di aderenza da parte degli operatori sanitari e non dell'Istituto nell'ambito della fase 1 della campagna vaccinale.

INFORMATIVA

L'Istituto Fiorentino di Cura e Assistenza (IFCA) S.p.A. è il promotore della survey. La survey prevede la consultazione dei dati di laboratorio, pseudonimizzati, relativamente alla rilevazione dei valori di Proteina S. Il questionario, in forma anonima, è somministrato in modalità cartacea a tecnici, amministrativi e sanitari operatori della struttura. I dati relativi alla Proteina S e il questionario saranno rivolti esclusivamente a operatori sanitari e non di IFCA. Il Promotore quale Titolare del trattamento dei dati La informa ai sensi e per gli effetti degli artt. 13 e 14 del regolamento UE 2016/679 de del d.lgs. 196/2003 che , in conformità con le responsabilità previste dalle linee guida di buona pratica clinica (D. Lgs n. 211/2003) Reg. Sper Clin, con il Regolamento UE 2016/679 Regolamento generale relativo alla protezione delle persone fisiche con riguardo al trattamento dei dati personali, nonché alla libera circolazione di tali dati (di seguito RGPD) e con il D. Lgs. 30 giugno 2003 Codice in materia di protezione dei dati personali recante disposizioni per l'adeguamento dell'ordinamento nazionale al regolamento (UE) n. 2016/679, tratterà i Suoi dati personali esclusivamente nella misura in cui siano essenziali per la conduzione della survey.

I dati, trattati mediante strumenti anche elettronici, saranno diffusi solo in forma rigorosamente anonima, ad esempio attraverso pubblicazioni scientifiche, statistiche e convegni scientifici. La pseudonimizzazione dei dati personali significa che i Suoi dati personali sono trattati in modo che non possano più essere attribuiti a Lei senza l'utilizzo di informazioni aggiuntive a condizione che tali informazioni aggiuntive siano conservate separatamente e soggette a misure tecniche e organizzative. I Suoi dati pseudonimizzati saranno conservati per un periodo non superiore a quello necessario per le finalità della survey per i quali sono stati raccolti - almeno 7 (sette) anni dalla conclusione della stessa. Il Titolare del trattamento per finalità sanitarie di cura, diagnosi e prevenzione e ai fini dell'esecuzione del presente Studio è il Centro presso il quale viene effettuata la survey. Per informazioni in merito al trattamento dei dati e per i diritti connessi, si prega di contattare il Responsabile della protezione dei dati del Centro di sperimentazione.

CONSENSO

- ☐ Dichiaro di aver letto l'informativa e presto il consenso alla partecipazione dell'indagine

Dichiaro di voler partecipare alla survey

- ☐ Si
- ☐ No

SURVEY
Risposta anticorpale e aderenza campagna vaccinale

Gent.ma / Gent.mo

1. Lei è un operatore:

   - ☐ Sanitario
   - ☐ Amministrativo
   - ☐ Tecnico

2. Lei ha effettuato la vaccinazione CIVID 19?

   - ☐ SI — Compilare "Sezione vaccinati" domande dalla 3 alla 7
   - ☐ NO — Compilare "Sezione NON vaccinati" domande dalla 8 alla 10

Sezione vaccinati

3. Prima di effettuare la vaccinazione aveva contratto il Covid-19?

   - ☐ SI
   - ☐ NO

4. Se si, quando ha contratto il Covid-19?

   Mese: ………………………. Anno: …………………………………

5. Ha completato la seconda somministrazione?

   - ☐ SI
   - ☐ NO

6. Quale vaccino Le è stato somministrato?

   - ☐ Pfizer
   - ☐ AstraZeneca
   - ☐ Moderna
   - ☐ Altro: …………….

7. A completamento della vaccinazione si è sottoposto al monitoraggio della Proteina S?

   - ☐ SI
   - ☐ NO

Sezione non vaccinati

8. Lei ha contratto fino a oggi il Covid-19?

   - ☐ SI
   - ☐ NO

9. Se si, quando ha contratto il Covid-19?

   Mese: ………………………. Anno: …………………………………

10. Ci può indicare la/le motivazione/motivazioni per cui non ha ancora effettuato il vaccino:

   - ☐ Indisponibilità di posti
   - ☐ Controindicazioni alla vaccinazione
   - ☐ Diffidenza sul vaccino
   - ☐ Preoccupazione effetti a lungo termine
   - ☐ Contagio precedente
   - ☐ Altro: ………………………………………………………………………
